# The molecular mechanism of action of methylene quinuclidinone and its effects on the structure of p53 mutants

**DOI:** 10.18632/oncotarget.26440

**Published:** 2018-12-14

**Authors:** Sara Ibrahim Omar, Jack Tuszynski

**Affiliations:** ^1^ Department of Oncology, Faculty of Medicine and Dentistry, University of Alberta, Edmonton, Alberta, Canada; ^2^ Department of Physics, Faculty of Science, University of Alberta, Edmonton, Alberta, Canada; ^3^ Department of Mechanical and Aerospace Engineering, Politecnico di Torino, Torino, Italy

**Keywords:** p53, methylene quinuclidinone, R175H mutant p53, R273H mutant p53, mechanism of action

## Abstract

One of the most important tumor suppressor proteins in eukaryotic cells is the transcription factor called p53. The importance of this protein in cells comes from the fact that it regulates a wide variety of cellular processes including the cell cycle, metabolism, DNA repair, senescence and apoptosis. In cancer cells, p53 is a major target as the most mutated protein, which has led to the search for potential activators of the mutant protein. Currently, the only mutated-p53 activator in clinical trials is a small molecule called APR-246. There is evidence that the active metabolite of APR-246 binds covalently to mutant p53 and restores its wild-type (wt) activity. In this work, we created atomistic *in silico* models of the wt, mutant and drugged mutant p53 proteins each in complex with DNA. Using molecular dynamics simulations we generated equilibrated models of the complexes. Detailed analysis revealed that the binding of the APR-246 active metabolite to the mutant proteins alters their interaction with DNA. In particular, the binding of the molecule at loop L1 of the protein allows the loop to anchor the protein to DNA similarly to wt p53. Several important p53-DNA interactions lost due to mutation were also restored in the drugged mutants. These findings, not only provide a possible mechanism of action of this drug, but also criteria to use in virtual screening campaigns for other p53 activators.

## INTRODUCTION

p53 is the master tumor suppressor protein [1–3]. It regulates diverse cellular processes including cell proliferation, apoptosis, senescence, metabolism and DNA repair [1–3]. While p53 is involved in several transcription-independent protein-protein interactions [4], it primarily mediates its activities by acting as a transcription factor that binds to p53 response elements to activate the transcription of canonical p53 target genes [3]. Given the vital importance of p53 in eukaryotic cells, especially its unequivocal tumor suppressor activity, it is not surprising that the p53 pathways are almost always disrupted in all types of cancers [5]. With a mutation rate of more than 50%, *TP53* is the most mutated gene in cancer [6]. These mutations often result in the loss of the tumor suppressor activity of p53 [7, 8]. The great importance of p53 in the context of cancer has made it an obvious but elusive target for anti-cancer treatment. Many strategies have been undertaken to reactivate the p53 pathways; one of these strategies is the restoration of the wild-type (wt) activity to mutant p53 (mp53) [1]. A few compounds have been identified to restore the wt activity to mp53 including PRIMA–1 (short for ‘p53 reactivation and induction of massive apoptosis’) [9], MIRA–1 [10], CP-31398 [11], 3-Methylene-2-norbornanone [12], STIMA–1 [13] and stictic acid [14].

APR-246, the methylated derivative of PRIMA–1, is the only mp53 activator that is currently in clinical trials [1, 15, 16]. A study by Lambert *et al.* [9] showed that PRIMA–1 and APR-246 are both prodrugs whose active product is methylene quinuclidinone (MQ). While it has been well-established that MQ restores the wt activity to mp53, additional mechanisms of MQ action, reviewed in [17], have been proposed. Nevertheless, MQ was primarily found to restore the transcriptional activity of mp53. This is supported by the fact that PRIMA–1-treated-mp53, transferred to p53 null cells, led to the activation of p53 target genes transcription and the induction of apoptosis [9]. Moreover, PRIMA–1 restores the correct folding of mp53 as evidenced by the binding of mp53 to wild-type p53 (wt-p53) conformation-specific PAb 1620 antibodies [9, 17, 18]. Also, differential scanning fluorimetry assays demonstrate that MQ increases the thermal stabilization of both G245S and R175H [14].

As mentioned above, MQ is the active product that reactivates mp53. MQ is a Michael acceptor, an α,β-unsaturated carbonyl compound, that reacts with and binds covalently to thiol groups in p53 increasing the mass of the protein and decreasing the percentage of its free thiols [9]. *In silico* modeling has shown that C124 is the most solvent accessible cysteine in p53 [14]. Furthermore, a pocket formed by loop L1 (residues 113–123) and beta-sheet S3 (residues 141–146), near C124, was found to transiently open during molecular dynamics (MD) simulations of the protein. Site-directed mutagenesis of C124 to alanine further confirmed the importance of this cysteine for the reactivation of mp53 by MQ [14]. The same study has also identified stictic acid as a p53 reactivator by virtually screening the NCI library at the C124 pocket [14].

We have previously docked MQ, NB, MIRA–1, STIMA–1, CP-31398, ellipticine, 9-hydroxy-ellipticine, WR–1065 and WR-2721 at the L1/S3 site near C124 [19]. As a result of this research, we have found that the reactive double bonds of the alkylating molecules MQ, NB, MIRA–1, STIMA–1 and CP-31398, are all directed towards the C124 thiol group in their best binding poses. However, ellipticine, 9-hydroxy-ellipticine, WR–1065 and WR-2721, which are non-alkylating p53 activators, were predicted to interact directly with C124.

Two of the highest frequency p53 mutant proteins, are R175H and R273H mp53, which differ from the wt protein sequence by a single missense mutation of the DNA binding domain arginine residues at positions 175 and 273 to histidine, respectively [7, 8]. The former p53 variant belongs to a class called structural mutants [20]. These proteins have a mutation in the DNA binding domain (DBD) residues, which do not directly interact with DNA yet cause structural unfolding, which prevents p53 from binding to its response elements [20]. The latter protein is classified under contact mutants, in which the mutation is in one of the residues that directly interact with DNA [20]. Although R175H and R273H mp53 are different types of mutants, which are structurally distinct [20], these two p53 variants are reactivated by MQ [9, 14].

Considering the unquestionable importance of p53 in maintaining and protecting the integrity of cells, it is disappointing that only one mp53 reactivator is currently in clinical trials. This fact has been attributed to the general perception that p53 is undruggable [1]. In this study, in order to challenge this perception we aim to understand the structural effect of the covalent binding of MQ to C124 of the two mp53 proteins. Moreover, we aim to understand if MQ alters structural (R175H-mp53) and contact (R273H-mp53) mutants differently. To this end, we have created equilibrated *in silico* atomistic models of the wt protein, the two mutants, as well as their ‘drugged’ forms in which MQ is covalently bound to residue C124 in the two mutants. The protein-DNA complexes were simulated for 750 ns to help achieve these goals. We analyzed the structures of these p53-DNA complex variants and compared them to the wt-p53-DNA complex. Important consequences emerged, which are discussed below.

## RESULTS

### p53-DNA complex structures

#### Mutant proteins

We used chain B of the p53-DNA complex 1TSR [21] PDB structure to create our models. R175H and R273H mp53 proteins were created by virtually mutating the arginine residues, at positions 175 and 273, to histidine using Pymol [22], respectively.

#### Drugged mutant proteins

We extracted the representative structures of R175H and R273H mp53 for the MD simulation times from 60 to 80 ns. We covalently docked MQ to C124 of the representative structures of the most populated clusters of each mutant. The covalent docking results for both R175H-mp53 and R273H-mp53 are described in Supplementary Table 1. The fact that MQ binds better to the less-populated clusters of mp53 can be attributed to the fact that the L1/S3 pocket (around C124) opens transiently [14]. The reaction of MQ's methylene with the sulphide of cysteine renders the reactive carbon of MQ chiral. Therefore, there are two possible modified-C124 epimers from this reaction, which we refer to as ‘CmQA’ and ‘CmQB’ in this article. The chosen poses from covalent docking were the drugged protein starting structures for our MD simulations.

**Figure 1:**
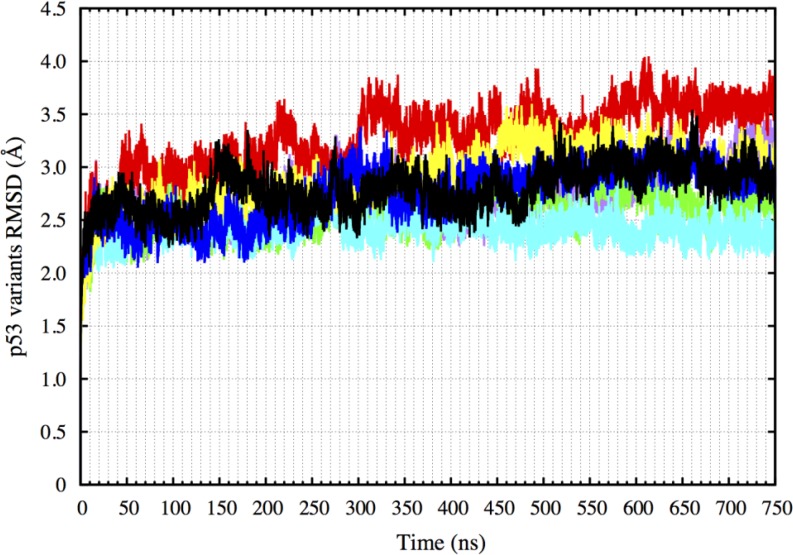
RMSD of the p53 variants non-hydrogen atoms over 750 ns The plot shows that all the p53 variants have equilibrated after 300 ns. Color scheme: wt-p53 (black), R175H-mp53 (red), R175H-CmQA-p53 (yellow), R175H-CmQB-p53 (blue), R273H-mp53 (cyan), R273H-CmQA-p53 (purple) and R273H-CmQB-p53 (green).

Following this, the wt-p53, R175H-mp53, R175H-CmQA-p53, R175H-CmQB-p53, R273H-mp53, R273H-CmQA-p53 and R273H-CmQB-p53 each complexed with DNA were simulated in explicit solvent using MD for 750 ns. These simulations are referred to as the original simulations in this article. We calculated the root-mean-square deviation (RMSD) of all the p53 variants’ non-hydrogen atoms over the course of the simulations to assess the proteins’ equilibration. Figure 1 shows that all the p53 variants have equilibrated after 300 ns of the simulation. This is evident by the plateauing in the RMSD values after 300 ns. All further analysis and comparisons reported in this article were performed on the last 450 ns of the MD simulations (from 300 to 750 ns).

Additionally, we ran 500 ns simulations of R175H-CmQB-p53 and R273H-CmQB-p53 DNA complexes as controls to assess the reproducibility of our results.

### Binding energy of p53 to DNA

#### Total binding energy

**Figure 2:**
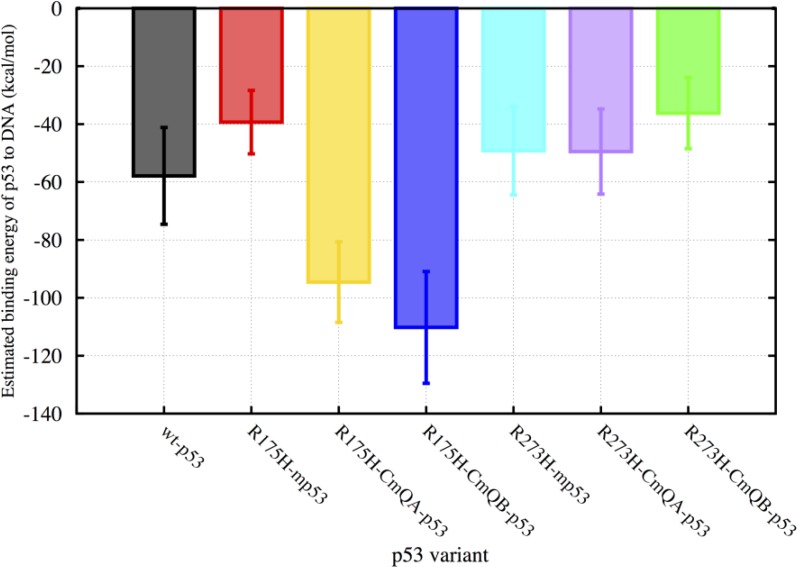
A bar graph of the EBEs of the p53 variants to DNA The binding energies of wt-p53, R175H-mp53, R175H-CmQA-p53, R175H-CmQB-p53, R273H-mp53, R273H-CmQA-p53 and R273H-CmQB-p53 to DNA were estimated using MMGBSA calculations from time 300 to 750 ns of the MD simulations. The error bars represent the standard deviation of the estimated binding energies during the simulation. The color scheme is the same as in Figure 1.

We used MMPBSA.py [23] in Ambertools to evaluate the binding energies of the p53 variants to DNA over the last 450 ns of the MD simulations (Figure 2). The calculated binding energies constituted the enthalpic and solvation energy contributions due to binding. Similar to our previous study [24], the change in conformational entropy due to binding was not included in our calculations; we refer to this calculated binding energy as the estimated binding energy (EBE). For the wt-p53, the EBE of the protein to DNA was −58 kcal・mol^−1^ with a standard deviation (SD) of 17 kcal・mol^−1^. The structural mutant R175H-mp53 had an EBE of -39 kcal・mol^−1^ (SD = 11 kcal・mol^−1^), which is almost 20 kcal・mol^−1^ more than the wt. For R175H-CmQA-p53 and R175H-CmQB-p53, however, the EBE was -95 (SD=14 kcal・mol^−1^) and −110 kcal・mol^−1^ (SD = 19 kcal・mol^−1^), respectively. This demonstrates that there was a marked increase in the affinity of the drugged *versus* undrugged structural mutants to the DNA.

The contact mutant, R273H-mp53, on the other hand, had an EBE of −49 kcal・mol^−1^ (SD = 15 kcal・mol^−1^) and its drugged variants, with the CmQA124 and CmQB124 residues, had EBEs of −49 kcal・mol^−1^ (SD = 15 kcal・mol^−1^) and −36 kcal・mol^−1^ (SD = 12 kcal・mol^−1^), respectively. It is expected that R273H-mp53 would have a lower affinity to DNA than wt-p53 since the native R273, which normally forms an electrostatic interaction with the DNA backbone, is mutated to the uncharged histidine residue. The EBEs of R273H-CmQA-p53 and R273H-CmQB-p53 indicate that the binding of MQ to R273H-mp53 does not increase the binding affinity of the modified protein to the p53 response elements. This is an indication that the gain in binding energy due to the mutation of arginine is not restored by the reaction of MQ with R273H-mp53. In fact, the mutant with epimer B has an even lower affinity to the DNA, −36 kcal・mol^−1^ (SD = 12 kcal・mol^−1^) compared to −49 kcal・mol^−1^ (SD = 15 kcal・mol^−1^) even when taking the standard deviation of the EBE into account.

#### Per-residue EBE

We further calculated the decomposition of the EBE per each residue of the complex to better understand the change in the interaction between the different p53 models and DNA. Figure 2 shows the contributions of the residues that had a lower EBE than −1 kcal・mol^−1^ or higher than 1 kcal・mol^−1^, for any of the p53 variants. Additionally, we also calculated the differences of these contributions between each residue of the p53 variants (ΔG_p53 variant res_) and the residues of wt-p53 (ΔG_wt-p53 res_) (Equation 1). These differences (ΔΔG_res diff_) were depicted on the p53 variants-DNA complex structures; the residues were colored as heat maps, ranging from blue (largest gain in EBE) to red (largest loss in EBE).

$$\Delta \Delta G_{resdiff}=\Delta G_{p53\mathrm{var}iantres}-\Delta G_{wt-p53res}$$ Equation 1

#### Mutants vs. wt-p53

**Figure 3:**
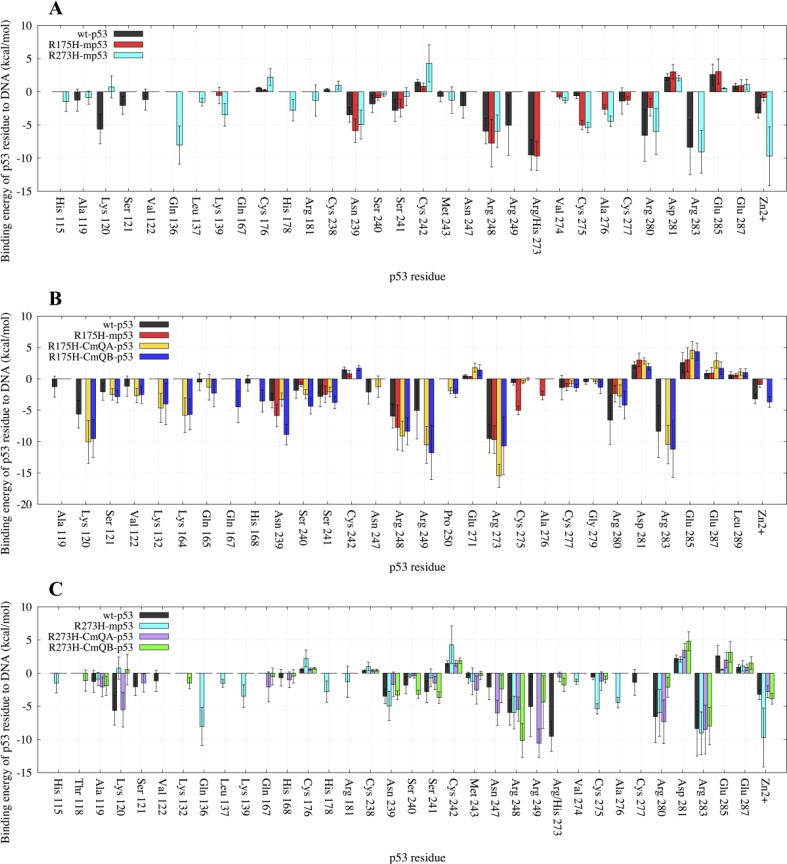
A bar graph of the decomposition of the EBE per-residue of p53 to the DNA Only the residues contributing more or less than 1 kcal・mol^−1^ to the EBE are shown. (**A**) Comparison between wt-p53 and the mutants: R175H-mp53 and R273H-mp53. (**B**) Comparison between wt-p53, R175H-mp53 and its drugged variants R175H-CmQA-p53 and R175H-CmQB-p53. (**C**) Comparison between wt-p53, R273H-mp53 and its drugged variants R273H-CmQA-p53 and R273H-CmQB-p53. The error bars represent the standard deviation of the EBE for each residue. The color scheme is the same as in Figure 1.

**Figure 4:**
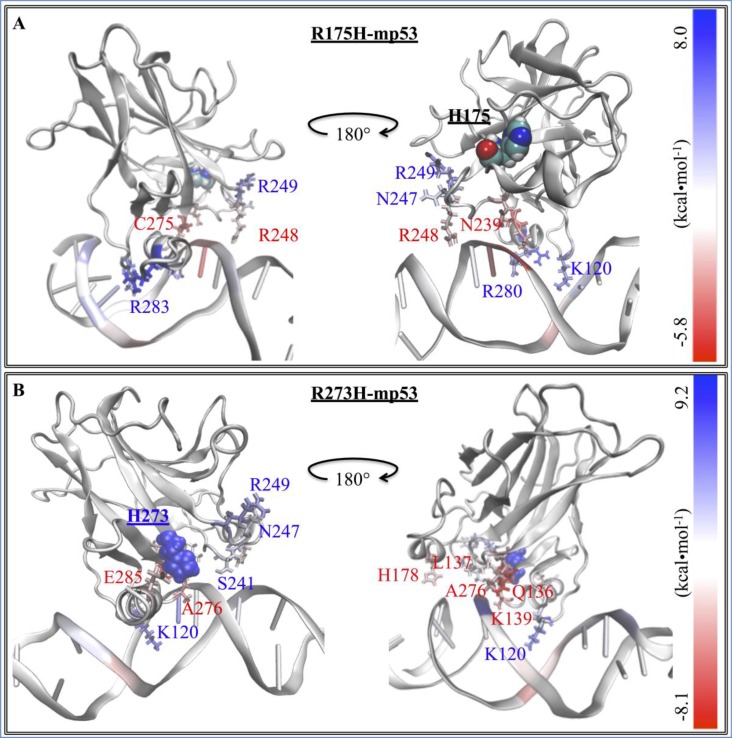
The difference in the EBE contribution of the mutant protein residues vs. wt-p53 A heat map representation of the difference between the EBE contributions of the residues (ΔΔG_res diff_) in the (**A**) R175H-mp53 and (**B**) R273H-mp53 vs. wt-p53 residues. The mutation sites are shown by their vdW representation. DNA interacting residues are shown by their line representation and color-coded according to their ΔΔG_res diff_, shown on the scale.

For the wt protein, Figure 3A shows that there were interactions between the DNA and R273, R283, R280, R248, K120, R249, N239, Zn^2+^, S241, N247, S121, S240, C277, A119, V122. C242, D281 and E285, in the order of increasing EBE. The structural mutant, R175H-mp53, which had a higher EBE to the DNA compared to the wt-p53 (−39 kcal・mol^−1^ vs. −58 kcal・mol^−1^), had fewer residues interacting with the DNA, namely: R273, R248, N239, C275, A276, S241, R280, C277, D281 and E285. Interestingly, some interactions with the DNA away from the mutated H175 residue were conserved in R175H-mp53, especially R273, R248 and N239. However, the interactions natively formed by M243 (albeit very weak) and N247, which are within 10 Å of the mutated H175, were completely lost (Figure 3A). Residues A119, K120, S121 and V122, which are in the L1 loop, also lost their interactions with the DNA in the structural mutant. Also, residues R249 and R283, which has the second highest affinity to DNA in wt-p53, completely lost their interactions with the DNA in R175H-mp53. Interestingly, A276 interacted with the DNA in R175H-mp53 but not in wt-p53. All other interactions between wt-p53 and the DNA were also present in R175H-mp53 and are within their standard deviation ranges with the exception of C275 of R175H-mp53, which was stronger in the structural mutant compared to the wt protein. A heat map of these interactions is shown in Figure 4A.

Although R273H-mp53 had a comparable EBE to DNA as the wt-p53, the contact mutant had more interactions with DNA. In the order of decreasing favorability, the residues contributing to the EBE for R273H-mp53 were: Zn^2+^, R283, Q136, R280, R248, C275, N239, A276, K139, H178, L137, H115, R181, V274, M243, E287, D281, C176 and C242. R273 had the highest affinity to DNA in the wt and R175H-mp53 models, with an EBE of about −10 kcal・mol^−1^ (Figure 3A). However, this interaction was completely lost in R273H-mp53 since the positively charged arginine residue is mutated to the neutral histidine, which also has a shorter side-chain. Figure 3A shows that the interactions with K120, S121 and V122 of loop L1 as well as N247 and R249 were diminished in R273H-mp53 compared to the wt protein. On the contrary, there were new interactions formed between the DNA and H115, Q136, L137, K139, H178, R181 and A276 of R273H-mp53 (Figure 3A). Like R175H-mp53, almost all the other interactions in wt-p53 were also present in R273H-mp53, within their standard deviation ranges, with the exception of C275, which is much stronger in R273H-mp53.

The interaction profiles of R175H-mp53 and R273H-mp53 with DNA suggest that these mutants have different binding poses to DNA compared to the wt-p53.

#### Drugged R175H-mp53 *versus* wt-p53

**Figure 5:**
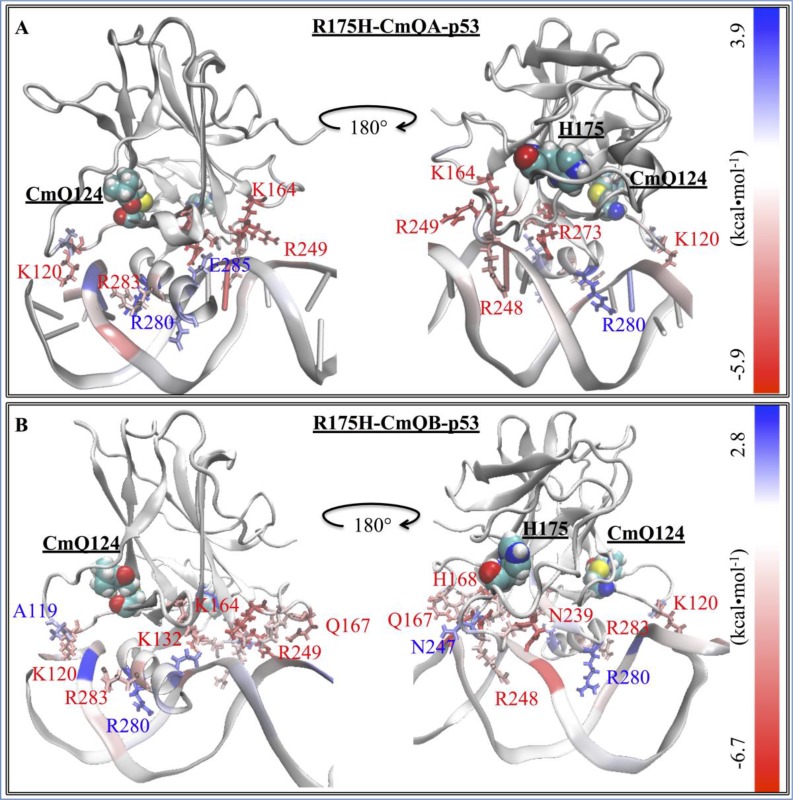
The difference in the EBE contribution of R175H-CmQA-p53 and R175H-CmQB-p53 vs. wt-p53 A heat map representation of the difference between the EBE residue contributions (ΔΔG_res diff_) of the (**A**) R175H-CmQA-p53 and (**B**) R175H-CmQB-p53 vs. wt-p53. The H175 and CmQ124 are shown by their vdW representation. DNA interacting residues are shown by their line representation and color-coded according to the EBE difference from wt-p53, shown on the scale.

R175H-CmQA-p53 and R175H-CmQB-p53 had an EBE of −95 and −110 kcal・mol^−1^, respectively, which was lower than the EBE of the wt-p53 to DNA (−58 kcal・mol^−1^). On further analysis of the interactions between the individual p53 residues and the DNA, Figures 3B and 5 revealed that both drugged mutant epimers, not only restored most of the lost interactions due to mutation, but also formed new interactions with the DNA.

Compared to R175H-mp53, the interactions of K120, S121 and V122 in loop L1, where the C124 MQ reaction site is also located, were restored in both drugged variants (Figure 3B). In addition, the strong interactions of R249 and R283, which had the second highest affinity to DNA in the wt-p53, were also restored. The interaction of Q165 in loop L2 was only present in wt-p53 and both drugged forms of the mutant protein. The relatively weaker N247 interaction present in the wt protein was also recovered in R175H-CmQA-p53. Also, the weak interaction of H168 with the DNA and wt-p53 was also present in R175H-CmQB-p53. It is most intriguing that both R175H-CmQA-p53 and R175H-CmQB-p53 also formed new interactions with DNA via K132, P250 as well as K164 in loop L2. Moreover, R175H-CmQB-p53 formed an additional new interaction through Q167.

However, the only residue whose interaction was not restored by either drugged form was a weak interaction of −1 kcal・mol^−1^ formed by A119 in the wt-p53-DNA complex. Interestingly, the interactions with DNA *via* residues C275 and A276, which were formed in R175H-mp53, were almost absent in the drugged structural mutants, like the wt protein.

#### Drugged R273H-mp53 vs. wt-p53

**Figure 6:**
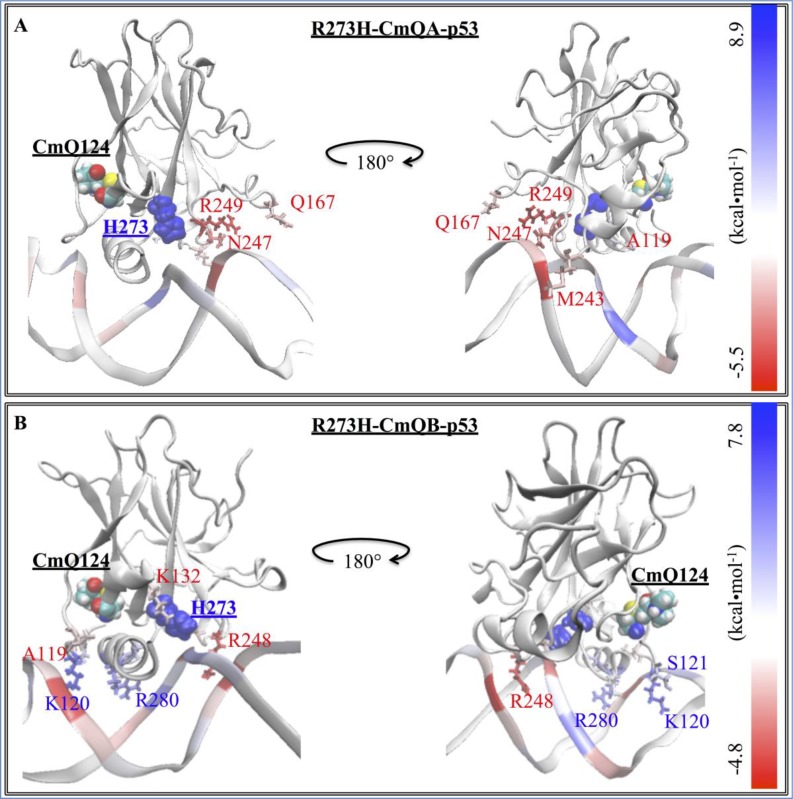
The difference in the EBE contribution of R273H-CmQA-p53 and R273H-CmQB-p53 vs. wt-p53 A heat map representation of the difference between the EBE contributions of the (**A**) R273H-CmQA-p53 and (**B**) R273H-CmQB-p53 residues vs. wt-p53. The H273 and CmQ124 are shown by their van der Waals (vdW) representation. DNA interacting residues are shown by their line representation and color-coded according to the EBE difference from wt-p53, shown on the scale.

The decomposition of the EBE for these variants are shown in Figure 3C and the heat maps of the complexes in Figure 6. Our models show that in both R273H-CmQA-p53 and R273H-CmQB-p53, the favorable interactions of R283, R280, R248, N239, A119 and M243 with the DNA were maintained, like the wt and the non-drugged contact mutant form. Figure 3C demonstrates that MQ binding restored the interaction of R249 and N247, like wt-p53, but not R273H-mp53, with the DNA. Also, R273H-CmQA-p53, like the wt, formed interactions with the DNA via K120 and S121 in loop L1. R273H-CmQB-p53, on the other hand, formed weak interactions with the DNA via residues T118. It is worth mentioning that R273H-CmQB-p53 also formed an interaction with K132, like the R175H-mp53 drugged forms, although it was stronger in the latter models. R273H-CmQA-p53, like R175H-CmQB-p53, also interacted with the DNA via Q167.

While our R273H-mp53 model interacted with the DNA via residues H115, Q136, L137, K139, H178, R181 and V274, none of these interactions existed in the drugged form of the mutant nor wt-p53. The drugged protein variants, however, unlike the wt protein, did not interact with the DNA via V122, C277 nor with the mutated H273 residue.

### Placement of the DNA

#### Alignment of the DNA to p53

**Figure 7:**
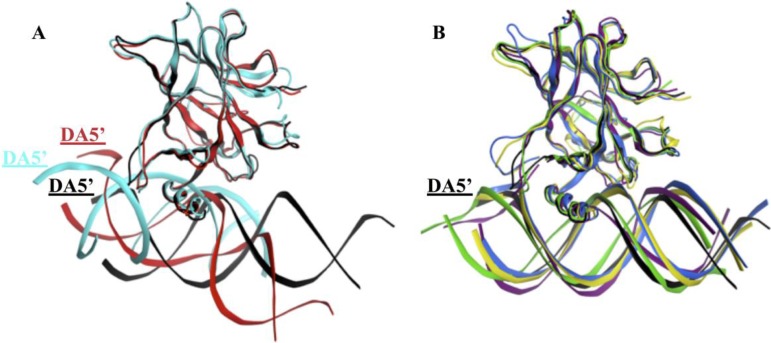
Superimposition of the p53 variants shows the displacement of the DNA in the mutants’ complexes (**A**) The DNA in the R175H-mp53-DNA and R273H-mp53-DNA complexes are displaced compared to the wt-p53-DNA complex. (**B**) The DNA molecules of the drugged mutants complexes were better overlaid with the DNA of the wt-p53. The color scheme is the same as in Figure 1.

We used Ambertools [25] to create the average structure of each p53-variant-DNA complex over the equilibrated part of the MD simulations (from 300 to 750 ns). We fitted the p53 variants’ backbone to the wt-p53 to compare the relative DNA positions of the different complexes. In Figure 7, the DNA of wt-p53 was horizontally on the plane, marked by its DA5’ end. However, the DA5’ ends of the DNA of R175H-mp53 and R273H-mp53 were projected in and out of the plane, respectively (Figure 7A). However, the superimposition of the drugged p53 variants average structures on the wt protein revealed that their complexed DNA stayed in the plane in a manner similar to that of the wt-p53-DNA (Figure 7B).

#### RMSD of the DNA

**Figure 8:**
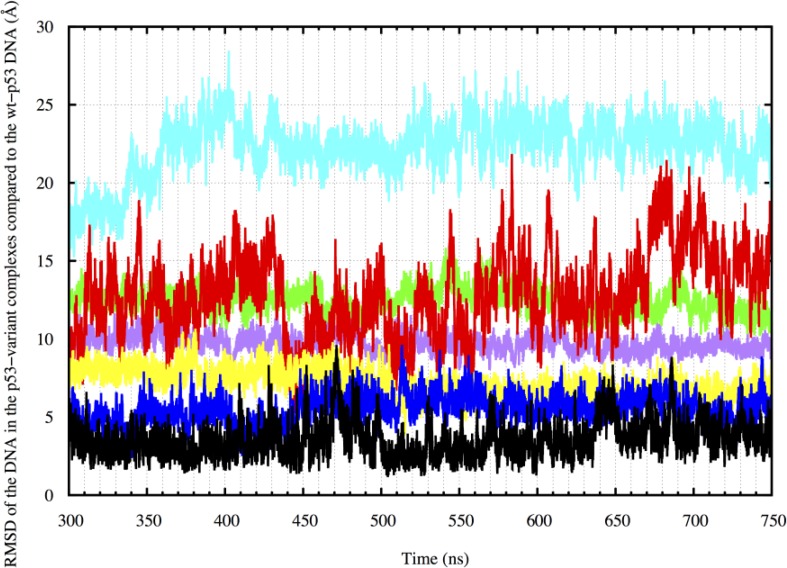
DNA RMSD in the p53 variant complexes compared to the wt-p53 DNA from 300 to 750 ns Wt-p53 has an RMSD of about 3 Å compared to its average structure. The drugged mutants have average RMSD values ranging from 9–15 Å. R175H-mp53 and R273H-mp53 on the other hand have average RMSD values of 18 and 20 Å, respectively. The color scheme is the same as in Figure 1.

We also calculated the RMSD of the DNA in the different complexes relative to the DNA in the average wt-p53 complex structure to assess the DNA alignment in a more quantitative manner. Figure 8 shows that the RMSD of the wt-p53 DNA had an average value of about 3.6 Å and reaches 9.6 Å during the simulation, relative to the wt-p53 average structure. The DNA of the structural mutant had average and maximum RMSD values of 12.8 and 21.9 Å, respectively, compared to the average wt-p53 DNA. Its drugged forms, on the other hand, had much lower RMSD compared to the mutants with average values of 7.2 Å and 5.5 Å for the ‘A’ and ‘B’ forms, respectively.

The DNA of R273H-mp53 had the highest RMSD from that of the wt with average and maximum RMSD values reaching 22.3 Å and 28.5 Å, respectively. Although both R273H-CmQA-p53 and R273H-CmQB-p53 had lower average RMSD of 9.7 and 12.4 Å, respectively, only the former lied within the RMSD ranges of the wt.

### RMSF of p53 residues

**Figure 9:**
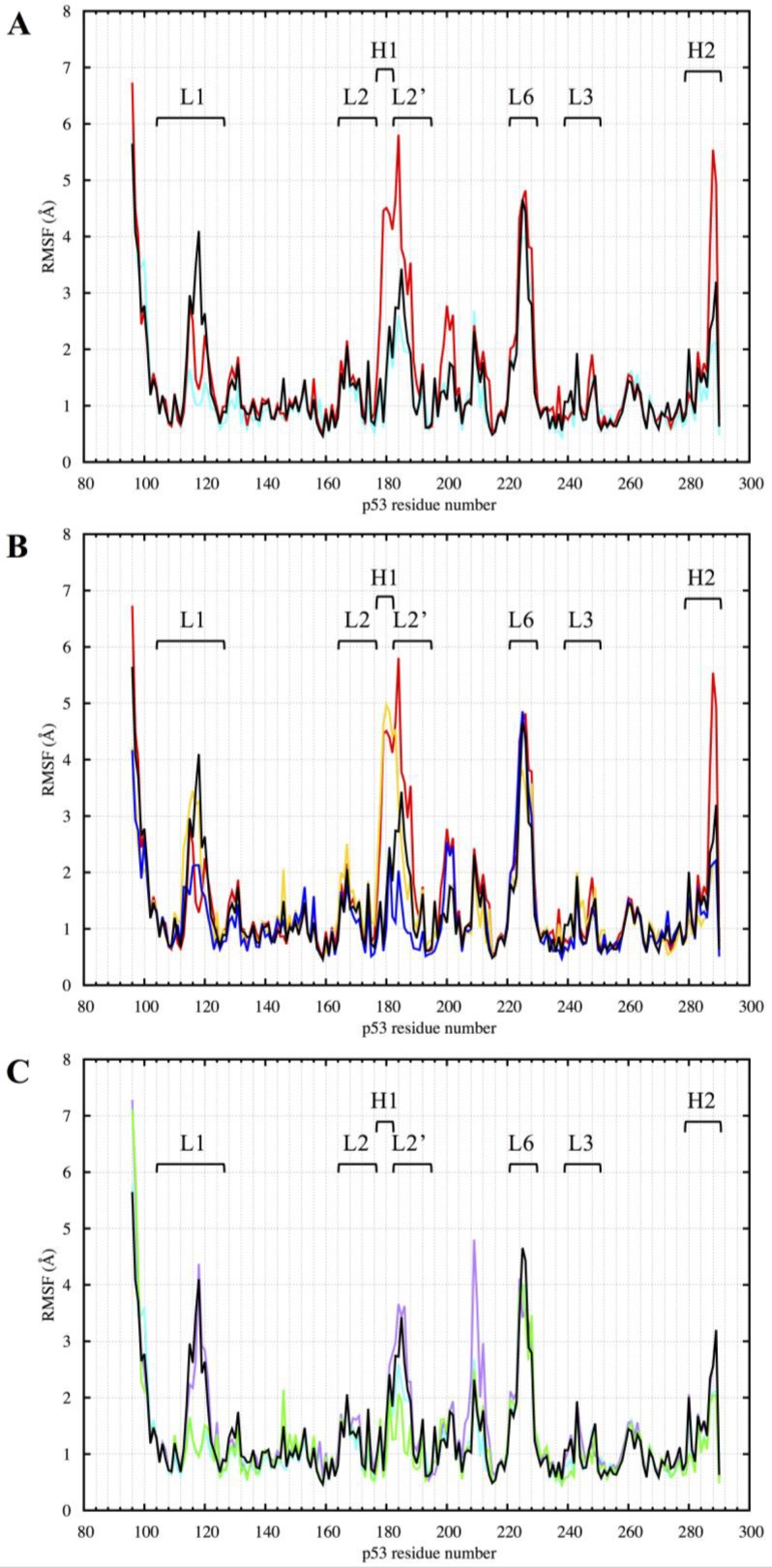
RMSF of the p53 variants DBD from 300 to 750 ns of the MD simulation (**A**) Comparison between R175H-mp53 and R273H-mp53 vs. wt-p53. (**B**) Comparison between R175H-mp53 and its drugged variants vs. wt-p53. (**C**) Comparison between R273H-mp53 and its drugged variants vs. wt-p53. Residue 290 is the Zn^2+^ ion. Marked are loops L1 (114–123), L2 (164–176, 182–194), L3 (237-250) and L6 (220-229) as well as helices H1 (177–181) and H2 (278-287). The color scheme is the same as in Figure 1.

We calculated the root-mean-squared-fluctuation (RMSF) of the protein residues in all the p53 variants over the equilibrated part of the MD simulations, from 300 to 750 ns (Figure 9). The coordinated zinc ions were assigned residue number 290 in each model.

#### Wt-p53

Most residues in the wt protein complex had low RMSF values reaching about 2 Å. The N-terminus residues had the highest RMSF, reaching more than 5.5 Å. However, the L1 loop, of which residues A119, K120, S121 and V122 are involved in DNA binding, had RMSF values reaching about 4 Å. On visually assessing this loop, our MD simulations showed that the L1 loop of wt-p53 visited two states known as the extended and recessed states. In the former state, K120 side chain is buried in the DNA major groove, while in the latter, the residue is out of the groove and interacts with the backbone phosphate of the DNA [26]. Another region with high fluctuations was loop L6, reaching more than 4.5 Å. This region is usually involved in the interface contact between the monomers of DNA-bound p53 dimers.

#### R175H-mp53 and its drugged variants

The R175H-mp53 structural mutant generally had the highest RMSF (see Figure 9A). This was specially observed for the L2 loop, where the R175H mutation lies. In addition, helix H2 had an RMSF that reaches about 5.5 Å compared to the wt, which had a maximum RMSF of about 3 Å for this region. Figure 9B shows that there were two distinct patterns for the fluctuations in R175H-CmQA-p53 vs. R175H-CmQB-p53. The latter was generally less fluctuating than its undrugged mutant, epimer A variant and the wt protein, especially near loop L2 close to the R175H mutation.

#### R273H-mp53 and its drugged variants

The p53 contact mutant had an RMSF pattern very similar to that of wt-p53 with only slightly lower RMSF, especially for the L1 loop residues (Figure 9A). Interestingly, there were also two distinct fluctuation patterns for the two drugged R273H-mp53 variants (Figure 9C). The RMSF pattern of R273H-CmQB-p53 was the same as that of R273H-mp53. However, the RMSF of R273H-CmQA-p53 closely resembled that of the wt-p53 except in residues 208 to 212.

A closer look at the fluctuations of epimers A *versus* B of both mutants revealed that p53 variants with the same epimers had similar RMSF patterns. This was demonstrated in Supplementary Figure 2A; the L1 loop of the drugged R175H-mp53 and R273H-mp53 with epimer A both had RMSF values reaching 4 Å as the wt-p53. However, R175H-CmQA-p53 had a higher RMSF than both the wt-p53 and R273H-CmQA-p53 in loop L2, where the R175H mutation lies. The resemblance between the RMSF patterns of R175H-CmQB-p53 and R273H-CmQB-p53 was more evident (see Supplementary Figure 2B), especially in loops L1 and L2, which had lower RMSF values than the wt protein.

### Control R175H-CmQB-p53 and R273H-CmQB-p53 simulations

**Figure 10:**
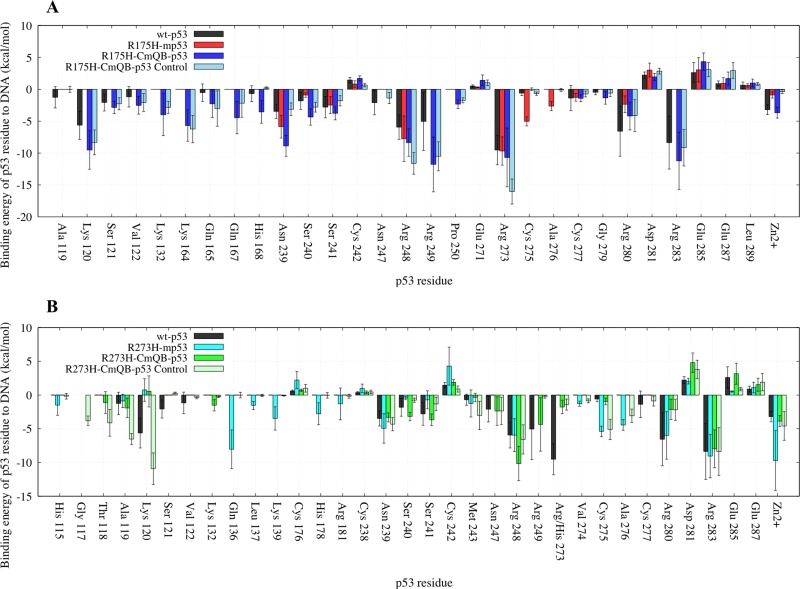
A bar graph of the EBE per-residue decomposition of p53 to DNA in the control simulations Only the residues contributing more or less than 1 kcal・mol^−1^ to the EBE are shown. (**A**) Comparison between wt-p53, R175H-mp53, R175H-CmQB-p53 (average structure from the last 450 ns of the 750 ns simulation) and R175H-CmQB-p53 (average structure from the last 100 ns of the 500 ns control simulation). (**B**) Comparison between wt-p53, R273H-mp53, R273H-CmQB-p53 (average structure from the last 450 ns of the 750 ns simulation) and R273H-CmQB-p53 (average structure from the last 100 ns of the 500 ns control simulation). The error bars represent the standard deviation of the EBE for each residue. Color scheme: wt-p53 (black), R175H-mp53 (red), R175H-CmQB-p53 (blue), R175H-CmQB-p53 from control simulations (light blue), R273H-mp53 (cyan), R273H-CmQB-p53 (green) and R273H-CmQB-p53 from control simulations (light green).

We also ran shorter 500 ns simulations of epimers B of the drugged variants. For these control simulations, analysis was performed on the last 100 ns. The EBE of R175H-CmQB-p53 and R273H-CmQB-p53 to DNA were −106 and −57 kcal・mol^−1^, respectively. The EBE decomposition per each residue of the control complexes *versus* the wt, mutants and epimers B of the drugged p53 variants from the longer simulations are shown in Figure 10.

The R175H-CmQB-p53 control variant had a very similar DNA interaction pattern as the same variant from the original simulations. The only exception was residue H168, which formed a stronger interaction in the original drugged variant, but not wt-p53. Also, N247 interacted with DNA in the control variant, like wt-p53, but not in the original R175H-CmQB-p53 complex (Figure 10A).

On the other hand, there were discrepancies between R273H-CmQB-p53 original and control variants (Figure 10B). More specifically, residues G117, T118, A119, K120, C275 and A276 interacted with the DNA in the control but not the original variants. Also, interactions between K132, S240, S241 and R249 of the original drugged variant were not reproduced in the control variant.

**Figure 11:**
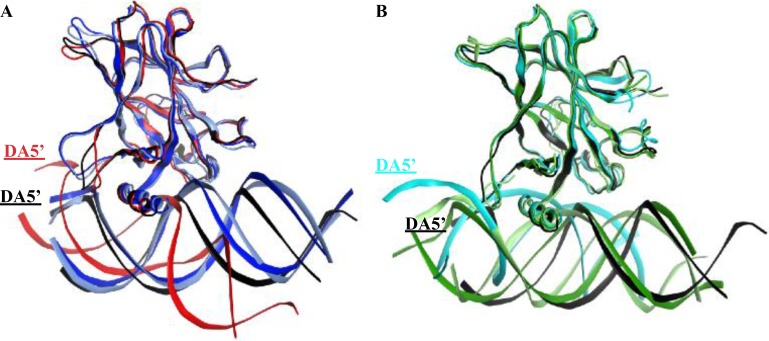
Superimposition of the p53 variants shows the displacement of the DNA in the mutants’ complexes and R273H-CmQB-p53 from the control simulations (**A**) The DNA in the R175H-CmQB-p53 complexes in both the original and control simulations are well overlaid with the DNA of the wt-p53 but not R175H-mp53. (**B**) The DNA molecule of R273H-CmQB-p53 control simulation is slightly displaced compared to the wt-p53-DNA complex. The color scheme is the same as in Figure 10.

Visual assessment of the DNA alignment in the control complexes average structures revealed that the DNA in the original and control R175H-CmQB-p53 complexes aligns well with the DNA in wt-p53. For the contact mutant drugged variants, however, the DNA in the control R273H-CmQB-p53 complex was less aligned with the DNA in wt-p53 than the DNA in R273H-CmQB-p53 from the original simulations (Figure 11).

## DISCUSSION

### Binding and alignment of p53 to DNA

We calculated the EBE of the p53 variants to DNA for the equilibrated portion of the MD simulation from 300 to 750 ns using the Molecular Mechanics Generalized Born Surface Area (MMGBSA) approach. Wt-p53 had an EBE of −58 kcal・mol^−1^ to DNA. Our calculations showed that both mutants, R175H-mp53 and R273H-mp53, had lower affinities to DNA with EBE of −39 and −49 kcal・mol^−1^, respectively. The R175H mutation is known to cause unfolding of the protein and a loss in its tumor suppressor ability [20] and hence the increase in EBE of this structural mutant is expected. Similarly, the increase in the EBE of the contact mutant is also expected but for a different reason: the native positively charged R273, which interacts with the backbone phosphate groups of the DNA in the wt protein, is mutated to the neutral histidine.

Our results showed that the reaction of MQ with C124 of the mutant proteins had different effects on their binding energies (Figure 2). The EBE of the drugged structural mutants to DNA were −95 and −110 kcal・mol^−1^ while those of the contact mutant were −49 and −36 kcal・mol^−1^. Collectively, these results indicate that the binding of MQ to the mutants induced a conformational change in the protein, especially that C124, the reaction site of MQ with p53, is not one of the DNA binding residues of p53. While the results for the drugged structural mutant indicate that MQ could be restoring the inactivity of mutated proteins by increasing their affinity, the drugged R273H-mp53 results showed that this was not always the case. In fact, the R273H-CmQB-p53 had an even lower affinity to DNA, namely 36 kcal・mol^−1^ (15 kcal・mol^−1^ SD) compared to the undrugged mutant, 50 kcal・mol^−1^ (12 kcal・mol^−1^ SD), even when taking the standard deviation of the EBE into account. It has previously been shown that the binding affinity of the wt-p53 was less than ten times stronger to its specific *versus* non-specific sequences [27, 28]. This small difference in affinities has shed light on the fact that the protein's affinity is not the only driving force for the recognition of p53 to its specific DNA sequences but rather its binding kinetics [28]. In fact, in a previous study, a designed S121F-V122G p53 double mutant-DNA complex had a life-time that was five-fold shorter than the wt-p53-DNA complex despite the fact that the designed double mutant had a binding affinity four times higher than that of wt-p53. This had lead us to examine other effects of MQ binding on the mutant p53 structure including assessing the individual residues that contribute to the binding energy.

#### Wt-p53

**Figure 12:**
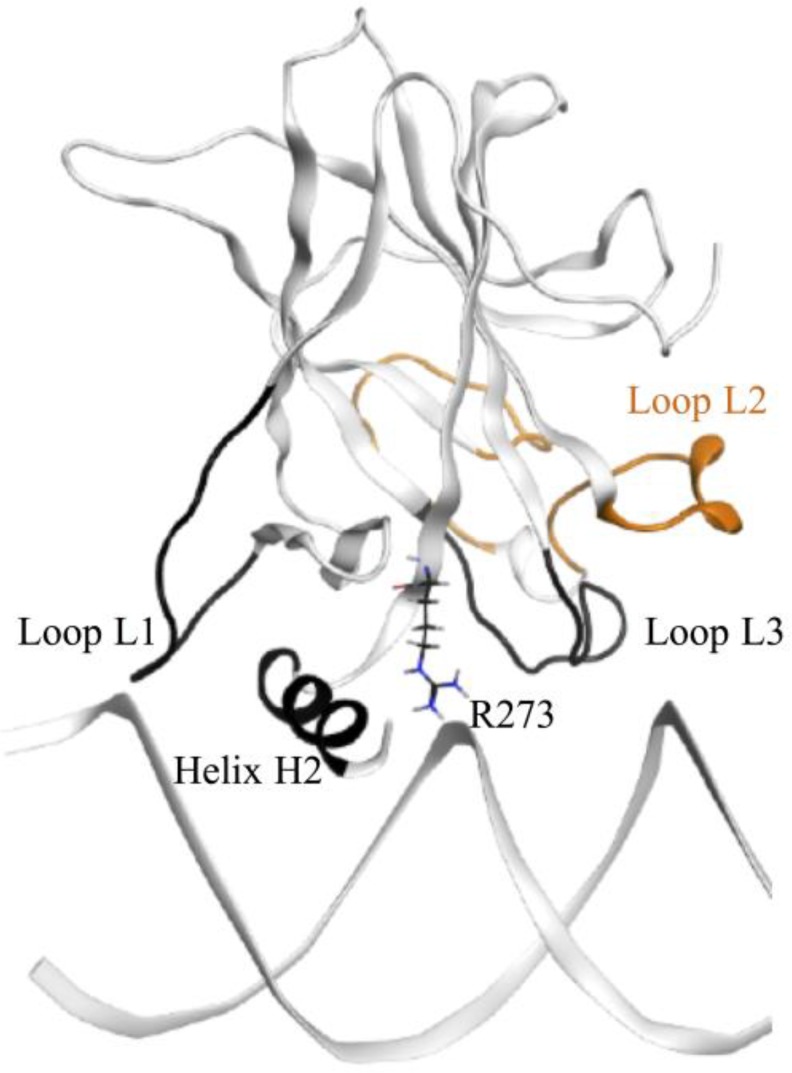
The wt-p53-DNA complex structure The L1 and L3 loops, helix H2 and R273, which form the main interactions with the DNA, are colored in black. They form a ‘base’, which sits on the DNA. Loop L2 is shown in orange.

Our wt-p53 model showed that the protein interacted with the DNA through loops L1, helix H2 (see Figure 3). A visual illustration of these p53 regions is shown in Figure 12. Residue R273, loops L1 and L3 as well as helix H2 can be seen as a ‘base’, by which the p53 sits on the DNA. Loop L1 can be considered the left side of this base. During our simulation, L1 loop of wt-p53 was mostly in its recessed form but also visited the extended state (Figure 12). This flexibility was reflected in the relatively higher RMSF of the L1 loop residues (Figure 9). Loop L3 can be considered the right side of the base, which interacts with the minor groove of the DNA (Figure 12). R273 and helix H2 form the center of the base (Figure 12); the latter interacted with the major groove of the DNA. It is through these interactions that the wt-p53 maintained its alignment with the DNA (Figure 7). Our models only constitute the DBD of the p53 variants. However, the fully functional p53-DNA complex is composed of a p53 tetramer. This tetramer is formed by both the interactions of the p53 tetramerization domains (not included in our models) as well as the interactions of the individual DBD with each other through residues in loop L6. It has been shown that p53 with a deleted tetramerization domain can both bind to DNA and possesses transcriptional activity [29]. Nevertheless, wt-p53 has a 100 times higher affinity to DNA as a tetramer than as a monomer [29, 30]. The symmetric alignment of p53 with the DNA is important to enable both the tetramerization of the protein and the cooperative binding of the DBD [31, 32].

#### R175H-mp53

This structural mutant is known to have a distorted conformational stability. The relatively higher RMSF values of the individual residues of R175H-mp53 confirmed this property (Figure 9). This was especially more pronounced in residue D184 in the L2 loop where the mutation site lies. On examination of the pairwise binding energy decomposition of R175 in wt-p53 vs. H175 in R175H-mp53, our results show that R175 formed electrostatic interactions with E180 and D184 during the simulation in wt-p53. In addition, R175 also formed electrostatic interactions with the Zn^2+^ coordinating H179. All these interactions were completely lost in R175H-mp53. Additionally, the mutation induced flexibility also caused the loss of coordination of Zn^2+^ by H179. Together, these local effects destabilized the mutant protein, especially at loop L2 as reflected in its high RMSF (Figure 9A). Visual inspection of the H2 helix during the simulation also revealed that the helix was partially unfolded towards its C-terminus and hence its high residue fluctuation.

Figure 3A demonstrates that the increase in the EBE of R175H-mp53 was due to the decrease in the number of residues that would normally interact with the DNA in the wt protein. Overall, it is evident that R175H-mp53 lost all its L1 loop interactions (the left side of the base) and three of eight loop L3 interactions (right side of the base) with the minor groove of the DNA (Figure 4A). We have previously used functional mode analysis to identify residues that correlate with the fluctuations in loop L3 [24]. Indeed, a correlation was found between the fluctuations in loops L2 and L3 as well as loops L3 and L1. This correlation can explain how a mutation in H175 (loop L2) can affect residues in loop L3, which subsequently affects residues in the L1 loop. Further, Figure 7 illustrates that R175H-mp53 did not maintain the same alignment pattern with DNA as the wt-p53. A more qualitative assessment of the DNA alignment is shown in Figure 8. The DNA of the mutant had an RMSD that exceeds 21 Å from the wt-p53. Additionally, the relative RMSD of the DNA in the structural mutant complex had the highest variation range from about 12.8 to over 21 Å.

Together, these results demonstrate that the R175H mutation caused a conformational change that altered the specific binding of p53 to the DNA response element. This could consequently contribute to the loss of the wt transcriptional activity of the mutant as well as its dominant negative effect since the mutant would not be able to form the symmetric tetramers around DNA.

#### R175H-CmQA-p53 and R175H-CmQB-p53

Both R175H-CmQA-p53 and R175H-CmQB-p53, not only restored most of the wt interactions with DNA lost due to mutation, but also formed new interactions with it. These interactions were reproduced in our control simulations for R175H-CmQB-p53 (Figure 10). As mentioned before, the wt protein interacted with DNA primarily through residues in loops L1 and L3 as well as through helix H2. Similarly, its drugged variants also maintained the same interactions except for A119. R175H-CmQB-p53 also lost the N247 interaction with the DNA.

Our models demonstrate that MQ binding to R175H-mp53 restored the L1 loop interactions (left side of the base) that were completely lost in the mutant protein. During our MD simulations, the L1 loops of both variants were in the recessed conformation but did not visit the extended conformation. This can explain the slightly lower RMSF of the L1 loop in both variants. Further on, the RMSF of the drugged variants, H175 of R175H-CmQA-p53 formed electrostatic interactions with D184 but not E180. There was, therefore, a slightly different RMSF pattern for that variant with E180 having the highest fluctuation in that region. Zn^2+^ was also not coordinated by H179 in R175H-CmQA-p53 probably due to the high fluctuation of its neighboring E180 residue. For R175H-CmQB-p53, on the other hand, H175 formed electrostatic interactions with E180, but not D184, which allowed the coordination of Zn^2+^ by H179. Hence the L2 loop in R175H-CmQB-p53 had a lower RMSF. Although the binding of MQ to R175H-mp53 seemed to at least partially restore the conformational stability of loop L2, the shorter mutated histidine residue side chain was still too short to interact with both E180 and D184.

The drugged structural mutants also formed additional interactions with DNA through their loop L2 residues, which do not usually do so in the native protein. These interactions seemed to have strengthened the right side of the base (Figure 5). Moreover, interactions in the L3 loop and H2 helix, which represent the right and center of the base, respectively, were also restored by MQ binding. It is worth mentioning that the unfolding of the H2 helix was not observed in the drugged variants, unlike R175H-mp53, as reflected in their RMSF. Overall, Figure 5 illustrates that both R175H-CmQA-p53 and R175H-CmQB-p53 lost some of the interactions in the wt-p53 with the DNA, yet maintained the base interactions with the DNA (red and white residues) that allowed it to align with the DNA in a manner similar to the wt (Figure 7B).

The RMSD of the DNA in both the R175H-CmQA-p53 and R175H-CmQB-p53 was higher than that of the wt protein's DNA to its average structure (Figure 8), yet still mostly lied below its maximum range. Nonetheless, the drugged variants had a much lower RMSD than their mutant form. Collectively, our findings indicate that MQ binding to R175H-mp53 did not restore the drugged mutant complex to become exactly like the wt-p53, yet the drugged complexes are structurally more similar to the wt protein than the mutant. The alignment of the proteins with the DNA also indicates that they would be more likely to form tetramers. The same alignment pattern was also observed in the R175H-CmQB-p53 control simulation (Figure 11).

#### R273H-mp53

In R273H-mp53, there was an expected loss in the interaction between H273 and DNA – the center of base. Further analysis indicated that this mutant had a different binding pattern to the DNA (Figure 3A). R273H-mp53 did not interact with DNA via loop L1 – left side of the base (Figures 3A and 4A). In fact, the L1 loop remained buried in the major groove of the DNA albeit at a different angle than the wt-p53. This had not allowed the same extended flexibility range for the loop and hence its lower RMSF (Figure 9A). Actually, R273H-mp53 generally had a similar or lower RMSF pattern to wt-p53 consistent with the fact that the R273H mutation is a contact one, which does not cause the unfolding of the protein. R273H-mp53 also formed weaker interactions with the DNA via its loop L3 representing the right side of the base, especially through residues N247 and R249 (Figure 4B).

Figure 7A shows that the loss in the base interactions of R273H-mp53 with DNA led to the loss of the protein's alignment with DNA, which was confirmed by the RMSD of the R273H-mp53 DNA relative to the wt-p53 DNA average structure (Figure 8). These findings can explain why this mutant loses its transcription ability in cells, since the misalignment of the protein with the DNA can hinder cooperative binding and tetramerization of the mutant.

#### R273H-CmQA-p53 and R273H-CmQB-p53

The reaction of MQ with R273H-mp53 did not improve the binding affinity of the protein. However, it changed the binding profile of the protein to the DNA. R273H-CmQA-p53 formed very similar interactions with the DNA like the wt-p53 especially in loops L1 and L3 as well as helix H2. This is evident in Figure 6A representing a heat map: the protein was mostly white (ΔΔG_diff_ = 0). The L1 loop interactions, which make the left side of the base were all restored except V122, which greatly deviated in the wt. Interactions via Q167 and H168 in the loop L2 were introduced, which made the right side of the base stronger. Additionally, the R249 interaction with the DNA minor groove, also making the right side of the base, was restored and even became stronger. It is evident from Figure 7B that R273H-CmQA-p53 had a similar alignment to the DNA like wt-p53 despite the fact that the interaction with residue 273 was not restored. This indicates that the right and left components of the base were enough to maintain the protein in the correct position relative to DNA. The RMSD of the DNA in this complex was closer to the wt than the mutant, although it was still higher than the latter. In addition, it was also similar to the R175H-mp53 drugged mutants, which have a higher affinity to DNA.

For R273H-CmQB-p53, several important interactions remained diminished, especially at the left of the base (Figure 6B). However, this drugged p53 variant still formed interactions through A119 and a new interaction through T118 in the L1 loop. In addition, our models showed that MQ binding to R273H-mp53 also restored the S240, S241, N247 and R249 interaction, which were lost in the mutant and constitute the center of the base. Although the binding pattern of R273H-CmQB-p53 did not show a strong binding profile like the other drugged variants, especially in the loop L1, it still seemed to form enough interactions for R273H-CmQB-p53 to maintain its alignment with DNA (Figure 7B). As mentioned above, R273H-mp53 did not align with DNA like the wt protein. It, therefore, formed unexpected interactions of beta-sheet sandwich residues with the DNA via Q136, L137 and K139. This was not the case in the drugged mutant variants. The qualitative assessment of the DNA RMSD in R273H-CmQB-p53 vs. wt-p53 showed that the RMSD of the former was closer to the DNA of the wt than R273H-mp53. Nonetheless, the deviation of the R273H-CmQB-p53 complex from the typical ‘base’ interactions was reflected in its DNA RMSD, which was the highest among all the drugged mutants (Figure 8).

Control simulations of R273H-CmQB-p53 further revealed a discrepancy in the interactions formed between R273H-CmQB-p53 in the control *versus* original simulations indicating that at least this drugged variant might not be activated by MQ, especially that the DNA in the control simulations did not well align with the DNA in the wt-p53.

As mentioned above, MQ has been previously shown to have other anti-cancer effects in cells [17]. It is possible that other mechanisms of MQ on other cellular targets cause the anti-cancer effect of MQ in treated cells carrying R273H-CmQB-p53.

On a general note, loop L6 of all the p53 variants was another region with high RMSF (Figure 9). Normally, the L6 loop residues are involved in p53 monomer-monomer interactions. Since our models represent a single p53 monomer bound to the DNA, it is expected that loop L6 would be more flexible and hence its high RMSF values in all the p53 variants. We have previously modeled wt-p53 apo monomers as well as a p53 dimer bound to the DNA. Evidently, the RMSF of loop L6 was indeed high in the apo monomers, however, it decreased in the p53 dimer bound to the DNA for the wt protein [24].

## MATERIALS AND METHODS

### Creating the p53-DNA complex models

#### Wild type p53-DNA complex

We used chain B of the 1TSR [21] X-ray determined structure of the wt-p53-DNA. We simulated this complex for 750 ns, using MD simulations (outlined below), to study the wt complex structure and compare it as a control to the mutant and drugged-mutant variants of the protein.

#### Mutant p53-DNA complexes

We virtually mutated the arginine residues at positions 175 and 273, in the chain B of the 1TSR wt-p53 structure, to histidines using Pymol [22] to create the starting complex structures of R175H mp53-DNA and R273H mp53-DNA, respectively. The chosen histidine rotamers were those that had the best fit to the structure, as scored by Pymol. Using the MD simulation protocol outlined below, the mutant complexes were simulated for 750 ns.

#### Drugged mutant p53-DNA complexes

We used CovalentDock [34] webserver to covalently dock MQ to C124 of both R175H-mp53 and R273H-mp53 (further explained below). Since the non-standard cysteine bound to MQ residue (CmQ) formed is a central peptide fragment, we created a dimethyl dipeptide of the non-standard cysteine fragment (Supplementary Figure 1) for parameterization. We used Gaussian [35] to optimize the geometry of the molecule and to derive its restrained electrostatic potential (RESP) charges with the HF 6-31G* basis set. We then used the PyRED webserver [36–39] to build the force-field libraries and parameters based on Amberff10, which is compatible with the more recent Amberff14SB. The parameterized drugged-mp53-DNA complexes were also simulated for 750 ns.

### MD simulations of p53

We used MOE [40] to protonate the p53-DNA complexes at pH 7, temperature of 310 K and 0.15 M salinity. The zinc ion coordinating C176, C238 and C242 were deprotonated. The parameters used for zinc ions were obtained from [41]. The protein-DNA complexes were all solvated in 12 Å TIP3P water boxes. Sodium ions were first added to neutralize the systems. Further, sodium chloride ions were then added randomly to reach a concentration of 0.15 M to simulate physiological conditions. We used AMBER [42] to run the MD simulations. The systems were minimized then gradually heated from 0 to 310 K with heavy restraints placed on the DNA and the protein backbone atoms. Before production, the restraints were gradually decreased until they were completely removed. The non-restrained systems were then simulated for 750 ns each at constant pressure and temperature of 310 K (body temperature). The SHAKE algorithm was also used to constrain the bond lengths to hydrogen atoms and enable the use of a 2 femtosecond timestep.

### Covalent docking

We used the CovalentDock [34] webserver to perform Michael addition covalent docking. To obtain the receptor structures, we clustered R175H and R273H mp53 from 60 to 80 ns in a manner similar to our previous work [19, 24]. We fit and clustered the p53 variants based on residues 114–117, 121–126, 133, 140–144, which surround C124. We used the representative structures of the most populated clusters for covalent docking. The DNA was removed from these structures and the mutant proteins were protonated in MOE [40]. C124 was set as the Michael addition site.

### Root-mean-square deviation and residue fluctuations

We calculated the root-mean-square-deviation (RMSD) of each complex during the simulation relative to its starting structure to assess the equilibration of the system using Ambertools’ cpptraj. We also calculated the RMSD values of the DNA in each complex relative to the wt-p53 DNA average structure by fitting the p53 variant structures to the backbone of wt-p53 average structure (from 300 to 750 ns). Since the N-terminal residues of the p53 DBD are flexible loops, we excluded the first 4 residues from the RMSD selection masks. We also calculated the root-mean-square-fluctuations (RMSF) of the p53 variants during the MD simulations.

### Binding energy calculations

The binding free energies of the equilibrated p53 variants to DNA were calculated using the Molecular Mechanics-Generalized Born Surface Area (MM-GBSA) in Ambertools [25]:

$$\Delta G_{bind}=\Delta H-T\Delta S\approx \Delta E_{MM}+\Delta G_{sol}-T\Delta S$$ Equation 2

In Equation 2, DG_MM_ is the molecular mechanic energy in gas phase, defined by the sum of the bond, angle, dihedral angles, electrostatic and van der Waals energies. DG_sol_ is the solvation free energy defined by the sum of the electrostatic and non-electrostatic components. The binding energies of the p53 variants to the DNA were calculated by Equation 3 as:

$$\Delta G_{bind,solv}^{{}^{\circ}}=\Delta G_{p53-DNA,vacuum}^{{}^{\circ}}+\Delta G_{p53-DNA,solv}^{{}^{\circ}}-\Delta G_{DNA,solv}^{{}^{\circ}}-\Delta G_{p53-p53,solv}^{{}^{\circ}}$$ Equation 3

## CONCLUSIONS

p53 is an architecturally fascinating molecule. Our models have shown that single point mutations at different sites of the protein can have profoundly different effects on the structure of the protein. Interestingly, the reaction of MQ at C124 with R175H-mp53 and R273H-mp53, which are essentially two different proteins especially in the way they interact with DNA, have one specific effect: they introduce interactions with DNA *via* loop L1. Since CmQ124 is not involved in direct interactions with DNA, this indicates that MQ could restore the transcriptional activity of the two mutants by inducing a conformational change in the protein. This conformational change seems to have led to the anchoring of p53 on the DNA, *via* loop L1, in a way that maintains the base interactions in the complex. This L1 anchoring was less pronounced in the original R273H-CmQB-p53 simulations, which indicates that that protein variant could be less active, especially that it had a much lower binding energy to DNA. However, control simulations of the same variant indicate that non-specific L1 loop interactions are also non-favorable since control R273H-CmQB-p53 interacted *via* G117, T118 with the DNA, yet did not well align with it like wt-p53.

The p53 mutants have been shown to have a lower binding energy to DNA, to form fewer interactions with it, especially the key base interactions and consequently are not aligned with DNA like the wt protein. The latter property is very important for the proper formation of p53 tetramers – the most transcriptionally active form of the complex. Site-directed mutagenesis studies have provided evidence that MQ covalently binds to p53 mutants at C124 [14]. However, a very recent study suggested that C277 was also key for reactivation of the mutants [33]. Our models provided evidence that MQ binding to C124 does indeed alter the binding of the drugged p53 mutants to the DNA. This is reflected in the restoration of key interactions with the DNA as well as the alignment of the proteins with the DNA in a manner more similar to wt-p53 than their mutants, from which the *in silico* models were built. Our results provide an understanding of the mechanism of action of MQ in the restoration of wt activity to mutant p53. This approach also provides a method of screening for potential p53 mutant activators that alter the protein structure, which is a very challenging task compared to screening for agonists or antagonists of a protein.

Since we do not observe a direct interaction pattern of MQ with p53, it is difficult to suggest specific recommendations for drug development of mp53 activators based on our models. Nonetheless, the work protocol used in this article could be used to assess the effect of potential activators on the interaction and alignment of p53 with DNA. Our study suggests that a successful mp53 activator would form the base interactions with DNA and align with DNA in a manner similar to wt-p53.

## SUPPLEMENTARY MATERIALS FIGURES AND TABLE


