# Supplementary material for: The molecular mechanism of action of methylene quinuclidinone and its effects on the structure of p53 mutants
Source: Oncotarget. 2018 Dec 14;9(98):37137–56. doi: 10.18632/oncotarget.26440 (PMC6324685; doi:10.18632/oncotarget.26440)
Supplement: Supplementary file 1 [file oncotarget-09-37137-s001.pdf]

The molecular mechanism of action of methylene quinuclidinone and its effects on the structure of p53 mutants

SUPPLEMENTARY MATERIALS

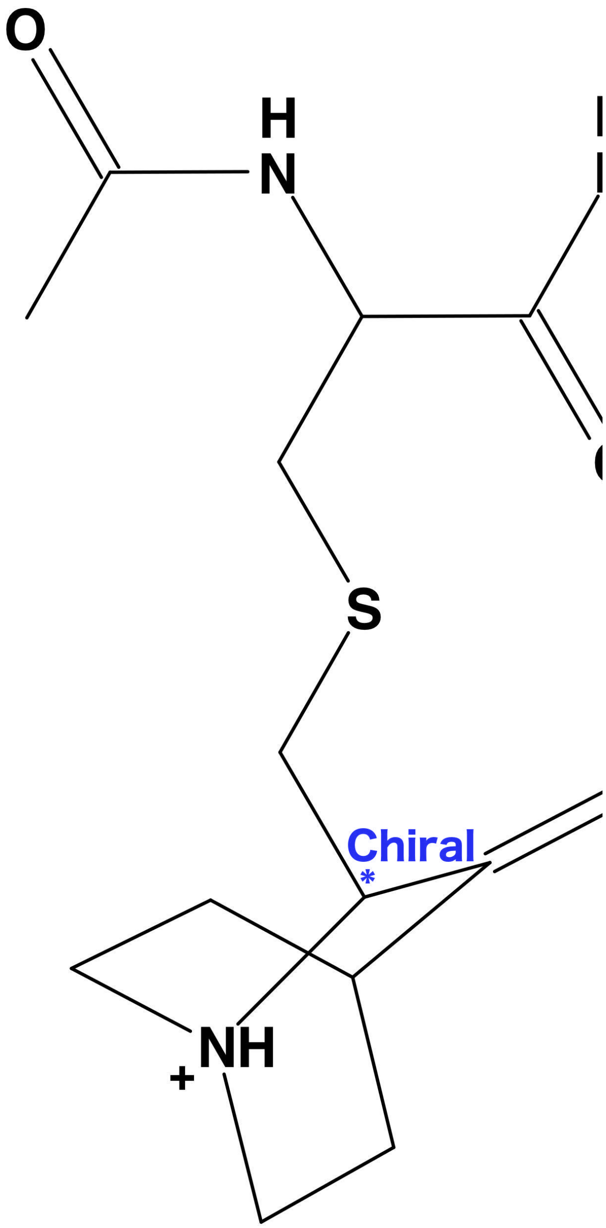

Supplementary Figure 1: Structure of the capped CmQ residue. CmQ is a modified C124 residue after the Michael addition reaction with MQ. The N-terminal cap is an acetyl group and the C-terminal cap is methylamine. The asterisk marks the chiral carbon, which leads to the formation of the A and B epimers of the molecule.

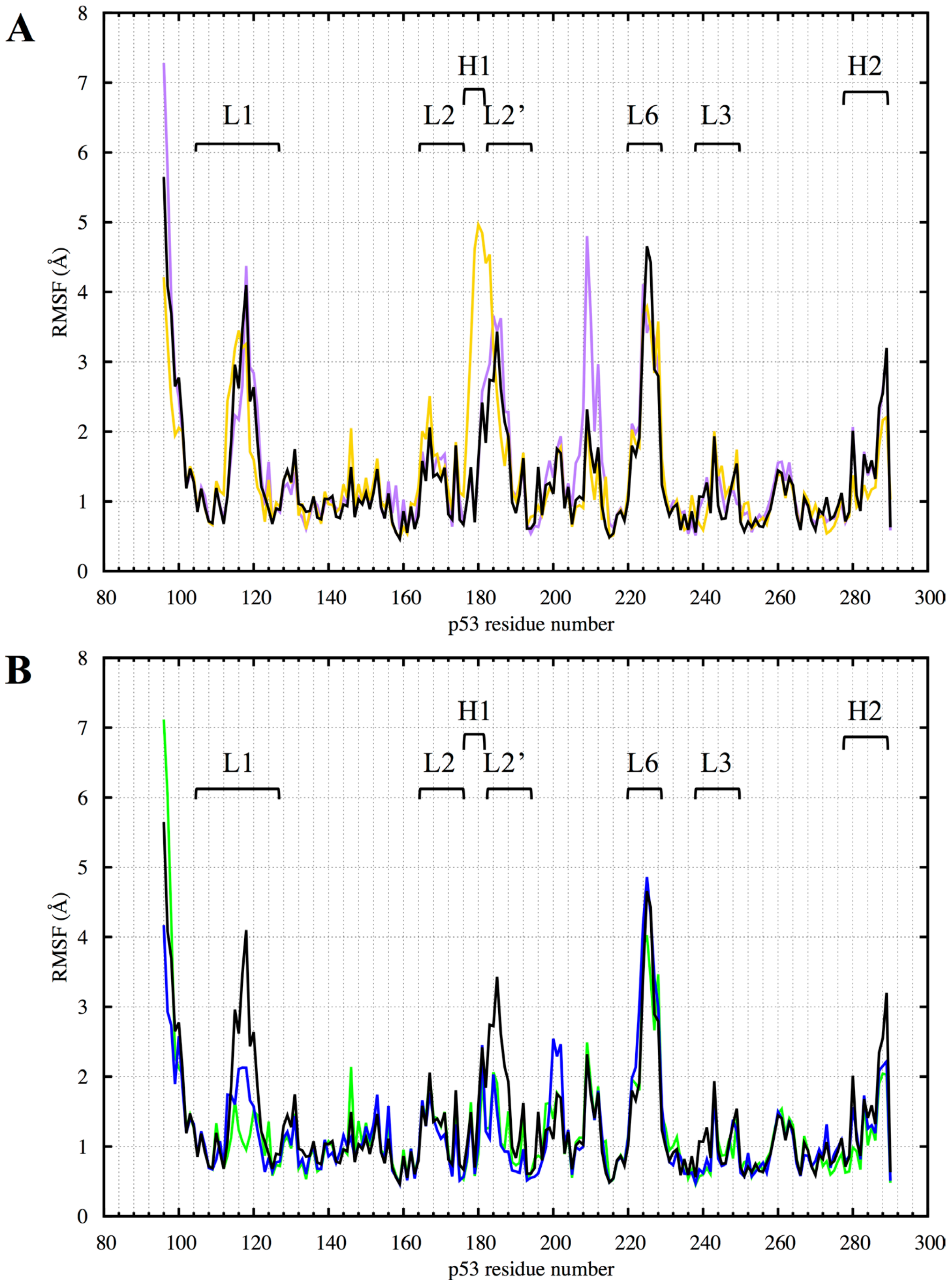

Supplementary Figure 2: RMSF of the p53 drugged mutants' DBD from 300 to 750 ns of the MD simulation. (**A**) Comparison between the RMSF of the DBD residues of R175H-CmQA-p53 and R273H-CmQA-p53 vs. wt-p53. (**B**) Comparison between the RMSF of the DBD residues of R175H-CmQB-p53 and R273H-CmQB-p53 vs. wt-p53. Residue 290 is Zn2+. Marked are loops L1 (114-123), L2 (164-176, 182-194), L3 (237-250) and L6 (220-229) as well as helices H1 (177-181) and H2 (278-287).

Supplementary Table 1: Covalent docking results of MQ to C124 of the representative structures of the two p53 mutants

Epimer	Lowest binding energy (kcal・mol^{-1})	Cluster size to which the best pose belongs	Number of clusters from docking
		R175H-mp53 representative structure 1	
A	−7.22	6	5
B	−7.76	5	3
		R175H-mp53 representative structure 2	
A	−7.48	10	1
B	−8.12	2	3
		R175H-mp53 representative structure 3	
A	−9.74	10	1
B	−10.03	10	1
	R273H-mp53 representative structure 1		
A	−9.73	8	2
B	−9.99	10	1
		R273H-mp53 representative structure 2	
A	−9.18	10	1
B	−9.50	10	1

CovalentDock webserver runs the docking calculation ten times. We chose the best binding pose to start our MD simulations based on the predicted binding energy and number of poses in the cluster. The docking results of the third representative structure of R175H-mp53 had the best-predicted binding energies for both epimers A and B and all the predicted poses belonged to the same docking bin. For R273H-mp53, the second representative structure from MD simulations had better docking cluster sizes than the first and their predicted binding energies were almost the same.
